# Recognizing early MRI signs (or their absence) is crucial in diagnosing metachromatic leukodystrophy

**DOI:** 10.1002/acn3.51692

**Published:** 2022-11-05

**Authors:** Daphne H. Schoenmakers, Shanice Beerepoot, Ingeborg Krägeloh‐Mann, Saskia Elgün, Benjamin Bender, Marjo S. van der Knaap, Nicole I. Wolf, Samuel Groeschel

**Affiliations:** ^1^ Department of Child Neurology, Amsterdam Leukodystrophy Center Amsterdam UMC location Vrije Universiteit Amsterdam, Emma's Children's Hospital Boelelaan 1117 Amsterdam The Netherlands; ^2^ Amsterdam Neuroscience, Cellular & Molecular Mechanisms Amsterdam The Netherlands; ^3^ Department of Endocrinology and Metabolism Amsterdam UMC location University of Amsterdam Meibergdreef 9 Amsterdam The Netherlands; ^4^ Center for Translational Immunology University Medical Center Utrecht Utrecht The Netherlands; ^5^ Pediatric Transplant Center Princess Máxima Center for Pediatric Oncology Utrecht The Netherlands; ^6^ Department of Child Neurology and Developmental Medicine University Children's Hospital Tübingen Hoppe‐Seyler‐Straße 1 72076 Tübingen Germany; ^7^ Diagnostic and Interventional Neuroradiology, Department of Radiology University Hospital Tübingen Hoppe‐Seyler‐Straße 3 72076 Tübingen Germany; ^8^ Department of Integrative Neurophysiology, Center for Neurogenomics and Cognitive Research Vrije Universiteit Amsterdam Amsterdam The Netherlands

## Abstract

**Objectives:**

Metachromatic leukodystrophy (MLD) has characteristic white matter (WM) changes on brain MRI, which often trigger biochemical and genetic confirmation of the diagnosis. In early or pre‐symptomatic disease stages, these typical MRI changes might be absent, hampering early diagnosis. This study aims to describe the characteristics of MRI WM abnormalities at diagnosis, related to clinical presentation.

**Methods:**

We retrospectively reviewed brain MRIs of MLD patients followed in 2 centers at the time of diagnosis regarding MLD MRI score and presence of tigroid pattern. In addition, MLD subtype, symptom status, CNS/PNS phenotype, motor/cognitive/mixed phenotype, and the presence of CNS symptoms were evaluated.

**Results:**

We included 104 brain MRIs from patients with late‐infantile (*n* = 43), early‐juvenile (*n* = 24), late‐juvenile (*n* = 20) and adult (*n* = 17) onset. Involvement of the corpus callosum was a characteristic early MRI sign and was present in 71% of the symptomatic late‐infantile patients, 94% of the symptomatic early‐juvenile patients and 100% of the symptomatic late‐juvenile and adult patients. Symptomatic early‐juvenile, late‐juvenile and adult patients generally had WM abnormalities on MRI suggestive of MLD. By contrast, 47% of the early‐symptomatic late‐infantile patients had no or only mild WM abnormalities on MRI, even in the presence of CNS symptoms including pyramidal signs.

**Interpretation:**

Patients with late‐infantile MLD may have no or only mild, nonspecific abnormalities at brain MRI, partly suggestive of ‘delayed myelination’, even with clear clinical symptoms. This may lead to significant diagnostic delay. Knowledge of these early MRI signs (or their absence) is important for fast diagnosis.

## Introduction

Metachromatic Leukodystrophy (MLD, OMIM 250100, 249900) is a lysosomal storage disorder with autosomal recessive inheritance. Its estimated incidence is 1:40.000 births, making it one of the most prevalent leukodystrophies.^1^ Based on the age of clinical manifestation, four different types of MLD can be identified: late‐infantile (<2.5 years), early‐juvenile (2.5–6 years), late‐juvenile (6–16 years), and adult MLD (>16 years).^2^ Biallelic pathogenic variants in *ARSA* or, in rare cases, *PSAP* result in decreased activity of arylsulfatase A,^3^, ^4^ an enzyme crucial for the lysosomal degradation of sulfatides. Consequently, sulfatides accumulate, particularly in membranes, which leads to demyelination. MLD, if untreated, results in relentless neurological deterioration and eventually premature death.^5^, ^6^ At present, there are only a few therapeutic options and those are restricted to pre‐ and early‐symptomatic patients: hematopoietic stem cell transplantation (HSCT) and autologous HSCT‐based gene therapy (GT). A trial for GT (NCT04283227) in late‐onset MLD is ongoing, as well as a trial for intrathecal enzyme replacement therapy (NCT03771898) in late‐infantile MLD. When the disease is too advanced, treatment is restricted to supportive care.^7^, ^8^, ^9^, ^10^, ^11^

Currently, newborn screening for MLD is under development and not yet widely available. Therefore, the diagnosis is usually made after clinical manifestation or through family screening. Brain MRI is an important diagnostic tool for leukodystrophies^12^ and guides biochemical and genetic testing. Characteristic MRI signs in MLD include bilateral T2‐signal white matter (WM) hyperintensities starting in the corpus callosum and expanding into the periventricular and central WM, with the subcortical WM being spared until late in the disease course. T2‐hypointense radially oriented stripes or dots on axial images, known as a tigroid pattern, are often present.^13^ Eventually, also the projection fibers and the cerebellum are involved, and signs of atrophy appear.^14^, ^15^, ^16^, ^17^ To quantify the severity of MRI abnormalities in MLD, a scoring system was developed, which has been widely used in the past decade.^18^ Biffi et al. (2008) adapted the MLD MRI score to increase its sensitivity, in particular for atrophy.^11^, ^19^

In MLD patients with a late‐onset form, brain MRI often shows extensive WM abnormalities at the time of diagnosis, whereas those with an early‐onset form can still have (almost) normal MRIs.^14^, ^16^, ^18^, ^20^ This implicates that around the time of diagnosis, just after clinical symptom onset, late‐infantile MLD patients may present with near‐normal MRIs, which hampers early diagnosis required for timely therapy. Therefore, we aimed to investigate the previously reported observations^14^ in a larger cohort and describe MRI changes for all four MLD subtypes in relation to clinical characteristics around the time of diagnosis.

## Methods

We conducted a retrospective study reviewing the medical records of patients followed at the Amsterdam Leukodystrophy Center (The Netherlands) or in the Tübingen University Hospital (Germany) up to January 10, 2022. Inclusion criteria were meeting the established genetic, biochemical, and clinical diagnostic criteria for MLD^21^; and an ‘early’ brain MRI with at least axial T2‐weighted or FLAIR sequences of sufficient quality. Early was defined in relation to the onset of first symptoms, using the earliest MRI available, defined as <1.5 years after symptom onset for late‐infantile, <2.5 years for early‐juvenile and late‐juvenile, and <5.5 years for adult patients (Box 1). Regarding pre‐symptomatic patients, there were no restrictions on time before onset. When a patient had more than one early MRI, the first scan was included. MRIs were visually assessed by a physician investigator (DS, NIW, SG) and the MLD MRI severity scores according to Eichler et al. (2009) were determined by a child neurologist experienced in using this score (NIW, SG). We used a previously established classification of MLD MRI scores, with scores ≤6 categorized as ‘mildly’, scores ≤15 as ‘moderately’, and ≥16 as ‘severely’ abnormal.^18^ In addition, the presence of a tigroid pattern was assessed. Mildly affected scans were visually compared with age‐matched controls and pre‐symptomatic scans.

Baseline patient and disease characteristics were collected. According to the age of symptom onset, patients were grouped into late‐infantile, early‐juvenile, late‐juvenile, and adult onset.^2^ The patients were categorized into pre‐symptomatic disease, defined as having no symptoms at all, and symptomatic disease. For all symptomatic patients, the phenotype was classified twofold as (1) having a motor, cognitive, or mixed phenotype, as we suggested earlier^5^, ^6^ and (2) having a CNS or PNS dominant phenotype. Definitions are described in Box 1. In addition, the presence and nature of initial misdiagnosis were evaluated.

Continuous variables were reported as mean, median and range. Ordinal variables were described using the median and interquartile ranges (IQR). Median MLD MRI scores were compared across subgroups of patients using the Mann–Whitney‐Wilcoxon Test or Kruskal‐Wallis multiple comparisons with Bonferroni correction as appropriate. Spearman's rank correlation was computed to assess the relation between the total MLD MRI severity score and the age at onset dependent subtypes, i.e., late‐infantile, early‐juvenile, late‐juvenile and adult MLD. All statistical tests were two‐tailed and *p*‐values below 0.05 were considered statistically significant. IBM SPSS Statistics 26 was used for all analyses and both IBM SPSS Statistics 26 and GraphPad Prism 9.1.0 (2021) were used for the creation of the figures.

## Results

### Overview patients

**Figure 1:**
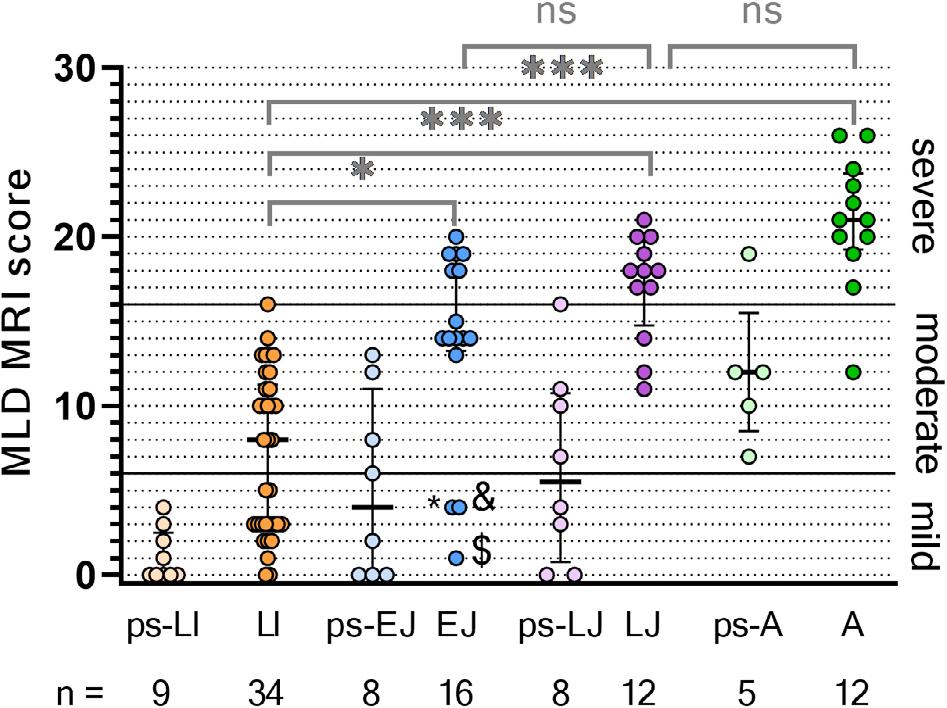
Clustered boxplot of total MLD MRI scores by MLD subtype. (ps‐)LI, (pre‐symptomatic) late‐infantile; (ps‐)EJ, (pre‐symptomatic) early‐juvenile; (ps‐)LJ, (pre‐symptomatic) late‐juvenile; (ps‐)A, (pre‐symptomatic) adult. * = early‐juvenile patient, 4.7 years old, age at onset 3.0, with PNS phenotype. & = early‐juvenile patient 4.2 years old, age at onset 3.5 years old, fine motor symptoms. $ = early‐juvenile patient 2.9 years old, age at onset 2.7 years old, reduced quality of walking, PNS phenotype. *p < 0.01; ***p < 0.0001; ns = not significant.

Forty‐three late‐infantile, 24 early‐juvenile, 20 late‐juvenile, and 17 adult patients fulfilled the inclusion criteria, resulting in 104 MRIs to be evaluated. Patient characteristics are shown in Table 1. The mean age at MRI was 1.9 years (median: 2.0; range: 0.4–2.9) for the 43 late‐infantile patients, 5.0 years (median: 5.5; range: 0.9–7.2) for the 24 early‐juvenile patients, 10.7 years (median: 9.3; range: 6.4–17.8) for the 20 late‐juvenile patients, and 26.1 years (median: 25.3; range: 17.0–36.3) for the 17 adult patients. There was a positive correlation between MLD MRI severity score and MLD subtypes, *r* = 0.58, *p* < 0.001, so later onset leads to higher scores. Symptomatic late‐infantile patients had significantly (*p* = 0.004, <0.001, <0.001 compared with early‐juvenile, late‐juvenile, and adult patients respectively) lower MRI severity scores and a wider distribution of the scores than the other symptomatic MLD subtype groups. (Fig. 1).

**Table 1:** Total number of patients.

	MLD subtype				
	Late‐infantile	Early‐juvenile	Late‐juvenile	Adult	Total
	*n* = 43	*n* = 24	*n* = 20	*n* = 17	*n* = 104
Age at MRI					
Mean (range)	1.9 (0.4–2.9)	5.0 (0.9–7.2)	10.7 (6.5–17.8)	26.1 (17.0–36.3)	
Sex					
Female	21 (49%)	8 (33%)	13 (65%)	10 (59%)	52 (50%)
Male	22 (51%)	16 (67%)	7 (35%)	7 (41%)	52 (50%)
Symptom status					
Symptomatic	34 (79%)	16 (67%)	12 (60%)	12 (71%)	74 (71%)
Pre‐symptomatic	9 (21%)	8 (33%)	8 (40%)	5 (29%)	30 (29%)
Motor, cognitive or mixed phenotype at onset					
Motor phenotype	30 (70%)	7 (29%)	5 (25%)	0 (0%)	42 (40%)
Cognitive phenotype	0 (0%)	0 (0%)	1 (5%)	8 (47%)	9 (9%)
Mixed phenotype	1 (2%)	7 (29%)	6 (30%)	4 (24%)	18 (17%)
PNS or CNS dominant phenotype at onset					
PNS	15 (35%)	5 (21%)	2 (10%)	0 (0%)	22 (21%)
CNS	12 (28%)	9 (38%)	10 (50%)	12 (71%)	43 (41%)
MLD MRI score					
Mild	25 (58%)	8 (33%)	4 (20%)	0 (0%)	37 (36%)
Moderate	17 (40%)	10 (42%)	6 (30%)	5 (29%)	38 (37%)
Severe	1 (2%)	6 (25%)	10 (50%)	12 (71%)	29 (28%)
Tigroid pattern					
Yes	16 (37%)	14 (58%)	13 (65%)	5 (29%)	48 (46%)
No	27 (63%)	9 (38%)	7 (35%)	5 (29%)	47 (45%)
Equivocal	0 (0%)	1 (4%)	0 (0%)	7 (41%)	8 (8%)

### Pre‐symptomatic patients

**Figure 2:**
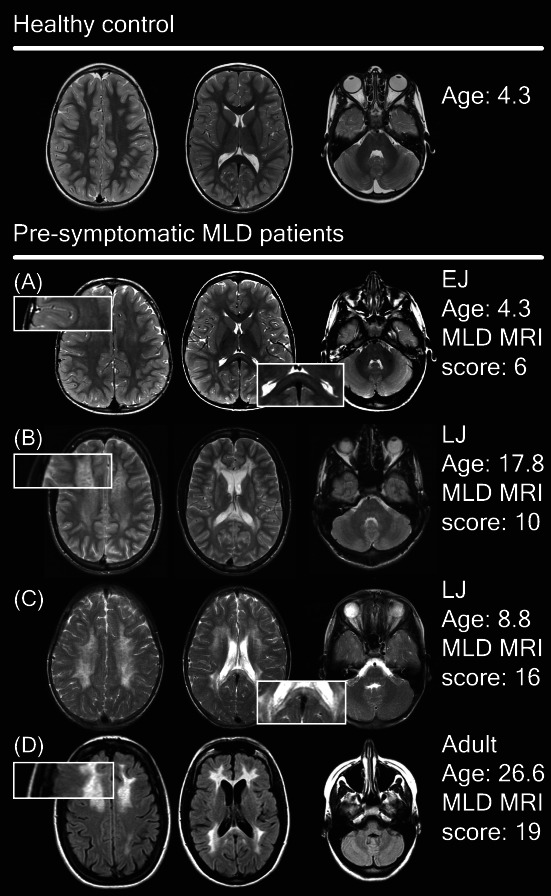
Spectrum of MRI abnormalities in pre‐symptomatic patients. (A) Faint T2‐hyperintensities in the splenium of the corpus callosum, the central and periventricular WM with sparing of the subcortical U fibers. (B and C) Much denser T2‐hyperintensities with a tigroid pattern. (D) Dense confluent frontal and parieto‐occipital hyperintensities on FLAIR images and signs of cerebral atrophy.

Pre‐symptomatic patients (*n* = 30) were typically scanned because of a positive family screening following the diagnosis of an affected sibling. Scans of late‐infantile patients varied from completely normal to mildly affected. (Figs. 1 and 2) In early‐juvenile and late‐juvenile patients, scans ranged from normal to severely affected. By contrast, MRIs from adult patients were all moderately or severely abnormal. In all MRIs of pre‐symptomatic patients with late‐infantile MLD the corpus callosum was completely normal, whereas the corpus callosum was involved in half of the pre‐symptomatic early‐juvenile patients, 75% of the late‐juvenile and all adult patients. None of the pre‐symptomatic late‐infantile patients presented with a tigroid pattern on their MRI. The minority of the MRIs of pre‐symptomatic early‐juvenile (*n* = 1, 13%), late‐juvenile (*n* = 2, 25%) and adult (*n* = 1, 20%) patients showed a tigroid pattern. An equivocal tigroid pattern was visible in one (13%) pre‐symptomatic early‐juvenile patient and in two (40%) pre‐symptomatic adult patients.

### Typical patterns in symptomatic patients

**Figure 3:**
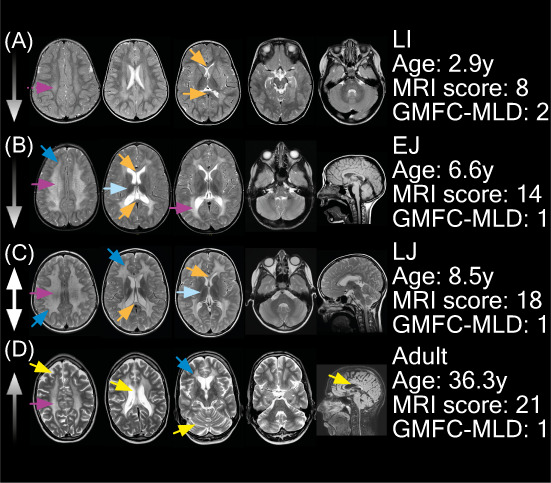
Typical MRI abnormalities in symptomatic MLD patients. Patient A had a PNS‐dominant motor phenotype, with CNS symptoms present. Patient B had a mixed phenotype with both motor and cognitive characteristics, but it was a PNS dominant phenotype. Patients C and D had a mixed phenotype, although these patients had a CNS dominant phenotype. Corpus callosum involvement; No (MLD MRI score ≤6); No (MLD MRI score >6); Yes (MLD MRI score ≤6); Yes No (MLD MRI score >6).

Figure 3 presents typical MRI abnormalities in symptomatic patients per MLD onset type. The median severity score per subtype (Fig. 1) was used to select a typical scan. In early stages, symptomatic late‐infantile MRIs showed faint confluent T2‐hyperintensities of the central and/or periventricular WM. The WM abnormalities were more or less symmetrically distributed, and the parieto‐occipital region was predominantly affected. In 24 (71%) MRIs of the symptomatic late‐infantile patients, the splenium of the corpus callosum was involved. A tigroid pattern was recognizable in 16 (47%) MRIs of symptomatic late‐infantile patients (Fig. 3). The subcortical U‐fibers were often spared. The internal capsule, corticospinal tracts, and cerebellar WM were normal at T2‐weighted images in nearly all patients.

**Figure 4:**
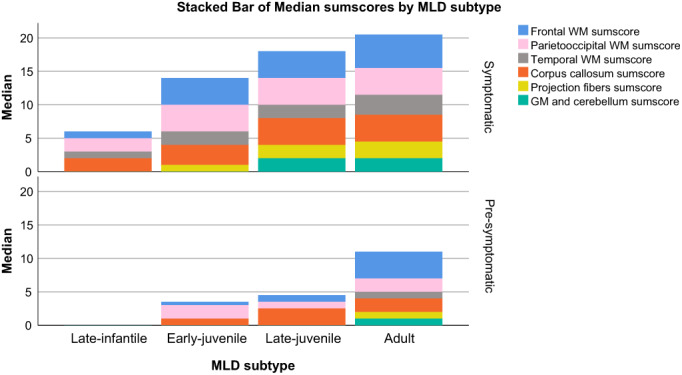
Stacked bar chart with median of MLD MRI sumscores. The median sumscores of grouped MLD MRI scoring items are visualized as stacked bar charts. The sumscores are composed as follows: frontal/parieto‐occipital/temporal WM sumscore = periventricular + central + U‐fibers; CC sumscore = genu + splenium; projection fibers sumscore = posterior limb of internal capsule + anterior limb of internal capsule + pons; gray matter and cerebellum sumscore = cerebral atrophy + thalamus + basal ganglia + cerebellum WM + cerebellar atrophy. The results were categorized based on MLD subtype and whether patients were pre‐symptomatic or symptomatic. Symp = symptomatic; Pre‐symp. = pre‐symptomatic; LI = late‐infantile; EJ = early‐juvenile; LJ = late‐juvenile; WM = white matter; GM = gray matter.

Symptomatic early‐juvenile MRIs typically revealed denser and more extensive T2‐hyperintensities than typical late‐infantile MRIs. The corpus callosum was involved in 15 (94%) patients. In 13 (81%) of the patients in this cohort a tigroid pattern was present. The parieto‐occipital regions were predominantly affected (Fig. 4). Symptomatic late‐juvenile MRIs generally had higher severity scores compared to early‐juvenile. In late‐juvenile MRIs, no clear frontal or parieto‐occipital predominance of abnormalities was recognized. A tigroid pattern was present in 11 (92%) symptomatic late‐juvenile patients. Symptomatic adult MRIs typically showed extensive signs of atrophy with for example enlarged peripheral cerebrospinal fluid (CSF) spaces, ventriculomegaly, thinned corpus callosum, and enlarged cerebellar sulci. Typically, the temporal brain regions were more affected than in the other MLD subtypes. No clear frontal predominance of WM abnormalities was observed in the MLD MRI severity score (Fig. 4). The corpus callosum was involved in 12/12 (100%) of the adult MRIs. A tigroid pattern was not (*n* = 3, 25%) or equivocally (*n* = 5, 42%) visible in most MRIs of adult patients.

### Clinical presentation

**Figure 5:**
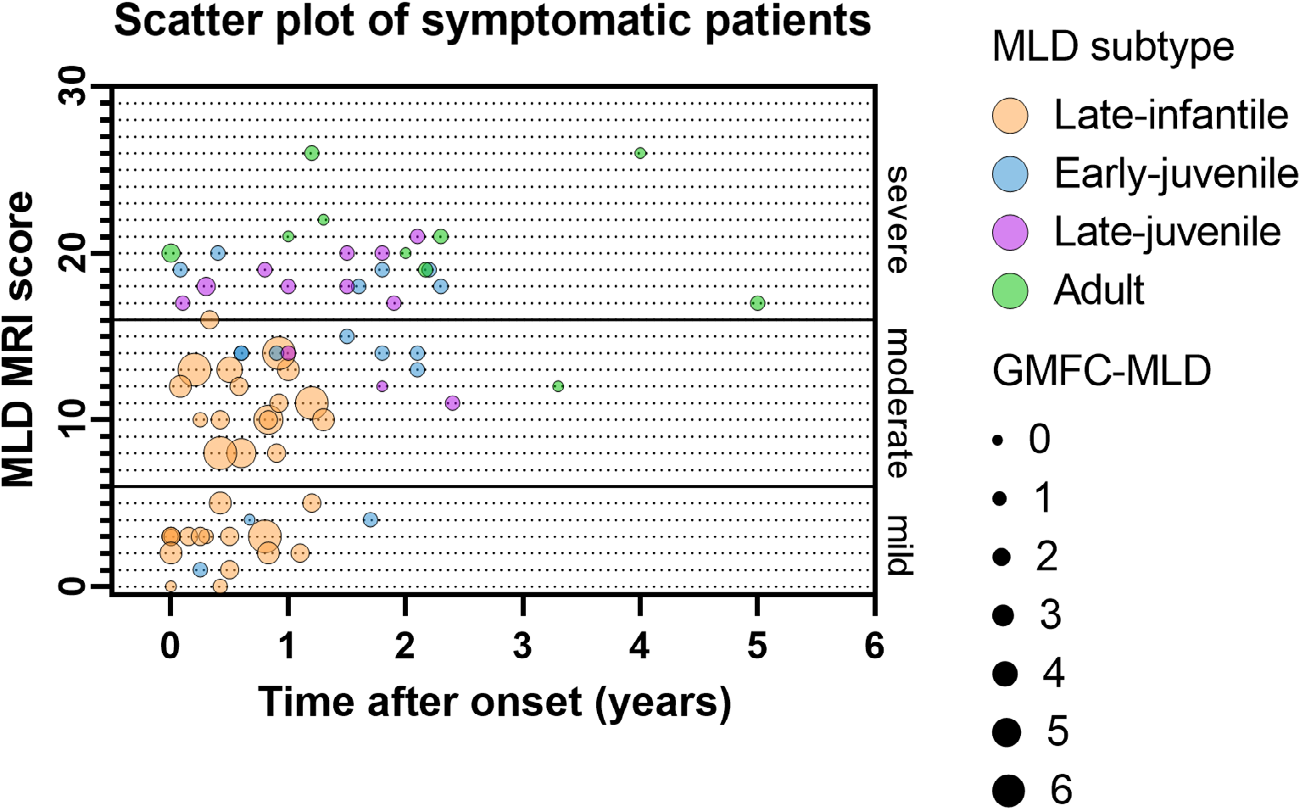
Scatter plot of MLD MRI score by time after onset and GMFC‐MLD.

Figure 5 presents gross motor function in relation to MLD MRI severity score and the time after onset. GMFC‐MLD scores in late‐infantile patients were generally higher than in the other patients and scores >1 could coexist with MLD MRI scores ≤6. In early‐juvenile, late‐juvenile, and adult patients GMFC‐MLD scores were often low (always ≤2), while MLD MRI scores were higher than in late‐infantile patients.

Symptomatic late‐infantile patients typically had a motor phenotype (*n* = 30, 88%), whereas early‐juvenile patients had a motor (*n* = 7, 50%) or mixed (*n* = 7, 50%) phenotype, late‐juvenile patients had a motor (*n* = 5, 42%), mixed (*n* = 6, 50%), or cognitive (*n* = 1, 8%) phenotype, and adult patients had a mixed (*n* = 4, 33%) or cognitive (*n* = 8, 67%) phenotype. Presenting with a cognitive or a mixed phenotype was associated with significantly higher MLD MRI scores (median: 21, IQR 20–23 and median: 17, IQR: 14–20, respectively) than a motor phenotype (median: 10, IQR: 3–13) with *p* values <0.001. Patients with a CNS dominant phenotype at onset generally had higher total MLD MRI scores (median MLD MRI score: 17; IQR: 13–20) than patients with a PNS dominant phenotype (median MLD MRI score: 10, IQR 3–12, *p* value <0.001).

### Two groups of symptomatic late‐infantile patients

About half of the symptomatic late‐infantile patients (*n* = 16, 47%) had a mild MLD MRI score, whereas the other half (*n* = 18, 53%) had moderate or severe MLD MRI scores. (Fig. 1) Patients with mild MLD MRI scores had a younger age at the time of the scan, a shorter time after onset, and generally a lower GMFC‐MLD score, as is shown in Table 2. Patients with MLD MRI scores above 6 tended to have more frequently CNS symptoms than patients with mild MLD MRI scores, although this difference was not significant (*p* = 0.078). No group differences were found in whether patients had a motor, cognitive or mixed phenotype or PNS or CNS dominant phenotype. The group of symptomatic late‐infantile patients with mild MRI involvement had evident symptomatology at the time of the scan, with GMFC‐MLD >1 in most cases (*n* = 12, 75%) and/or clear CNS symptoms (*n* = 10, 63%).

**Table 2:** Symptomatic late‐infantile patients.

	MLD MRI score		*p* value
	0–6	> 6	
	(*n* = 16)	(*n* = 18)	
Mean age at onset (range)	1.5 (1.0–2.5)	1.6 (1.0–2.3)	0.251
Mean age at scan (range)	1.9 (1.2–2.4)	2.3 (1.8–2.9)	<0.001*
Mean time after onset in years (range)	0.4 (0.0–1.2)	0.7 (0.1–1.3)	0.030*
Corpus callosum involvement			
Yes	6 (38%)	18 (100%)	<0.001*
No	10 (63%)	0	
Median GMFC‐MLD score at the time of MRI	2	3	0.025*
Motor, cognitive or mixed phenotype at onset			
Motor phenotype	15 (94%)	15 (83%)	0.516
Cognitive phenotype	0	0	
Mixed phenotype	0	1 (6%)	
Missing	1	2	
Presence of CNS symptoms, e.g. pyramidal tract signs or cognitive decline			
Yes	10 (63%)	16 (89%)	0.078
No	3 (19%)	0	
Missing	3	2	
PNS or CNS dominant phenotype at onset			
PNS	6 (38%)	9 (50%)	0.619
CNS	5 (31%)	7 (39%)	
Missing	5 (31%)	2 (11%)	

* *p* ≤ 0.05, *p* values for mean age at onset, mean age at scan, mean time after onset, and GMFC‐MLD with Mann–Whitney U, other *p* values with Fisher's Exact test.

**Figure 6:**
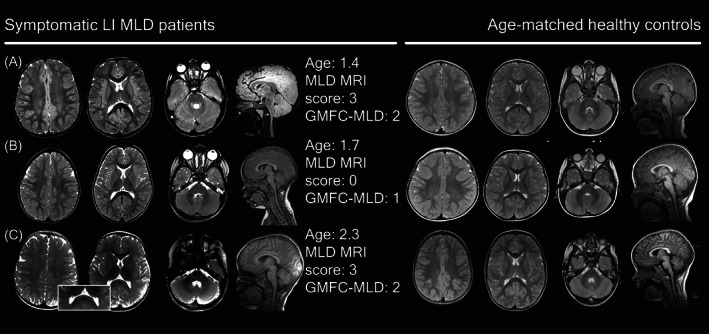
Less typical MRIs in symptomatic late‐infantile patients compared to healthy controls.

**Figure 7:**
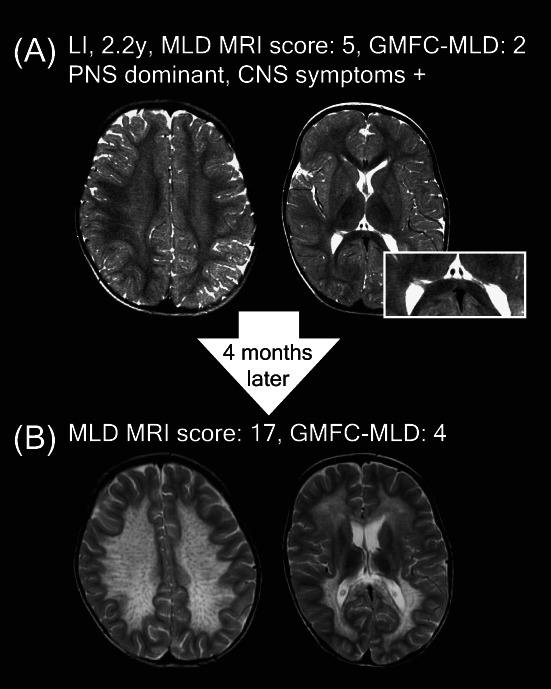
Follow‐up of a symptomatic late‐infantile patient.

Some of the mildly affected MRIs of symptomatic late‐infantile patients had corpus callosum involvement (*n* = 6, 38%), whereas none had a tigroid pattern. When comparing those mild MRIs with age‐matched controls, suspicious age‐inappropriate T2‐hyperintensities, in particular in the central WM could be recognized. In early stages, small subtle T2‐hyperintensities located in the mid‐splenium could correspond to beginning demyelinating lesions (Figs. 6 and 7A). Interestingly, corpus callosum abnormalities were absent in patients with an age below 1.75 years. Mild late‐infantile scans differed from mild pre‐symptomatic juvenile scans because there was a higher contrast in signal intensity between the faint WM hyperintensities and the spared subcortical U‐fibers in the patients with juvenile onset (Fig. 2A).

These very mild late‐infantile MRIs can, however, rapidly progress to moderate or severe MRIs, as shown in the example of a 2.2‐year‐old late‐infantile patient who presented with stagnation of motor developmental and subsequent regression after a flu‐like illness, with an eye movement disorder, severe hypotonia, and areflexia, indicating a PNS dominant phenotype. The MRI showed subtle T2‐hyperintensities (Fig. 7A) leading to initial misdiagnosis with chronic inflammatory demyelinating polyneuropathy. Four months later, the MRI had deteriorated from MLD MRI scores 5 to 17, with a now typical MLD pattern (Fig. 7B) In hindsight, a subtle splenial lesion was already present on the initial MRI (Fig. 7A).

### Initial misdiagnosis

In total, 10 late‐infantile patients were initially misdiagnosed with chronic inflammatory demyelinating polyneuropathy (*n* = 4), unclassified polyneuropathy (*n* = 1), Déjérine‐Sottas Disease (*n* = 1), Segawa syndrome (*n* = 1), oculomotor apraxia (*n* = 1), post infectious gait ataxia (*n* = 1), or spinocerebellar ataxia (*n* = 1). In all these cases, a normal appearing MRI was an important factor for a diagnosis other than MLD. Half of these patients had a PNS dominant phenotype, but in 9/10 patients, pyramidal tract signs pointing to CNS involvement were already present.

## Discussion

The typical MRI abnormalities of MLD reflecting diffuse symmetrical demyelination are well‐known and usually prompt diagnosis of this leukodystrophy. In this study, we aimed to describe early MRI findings around the time of diagnosis and the corresponding clinical presentations. In nearly all symptomatic early‐juvenile, late‐juvenile, and adult patients in our cohort, typical MLD patterns, as reported in several previous studies,^11^, ^12^, ^13^, ^14^, ^17^, ^22^, ^23^ could be recognized. Splenial corpus callosum demyelination was found to be a sensitive MRI sign, present in nearly all those MRIs. A tigroid pattern, which is considered characteristic for lysosomal storage disorders,^13^ was present in most MRIs of juvenile patients, whereas this pattern was not easy to recognize in the hyperintense WM in MRIs of patients with adult‐onset MLD. The high MRI scores found in the latter group are consistent with previous studies.^18^, ^24^

In contrast, about half of the symptomatic late‐infantile patients had no or mild MRI abnormalities, even in the presence of clinical CNS involvement, with scans in this group being either normal or only showing faint T2‐hyperintensities in the centrum semiovale, central WM, or the corpus callosum. However, in 31% of those mildly affected late‐infantile and 100% of the moderately affected late‐infantile scans, the splenium of the corpus callosum was involved, underlining the importance of recognizing this structure as an important early MRI sign for late‐infantile MLD. Thus, we could substantiate our earlier findings that in late‐infantile MLD MRI abnormalities gradually evolve after symptom onset while in juvenile MLD MRI abnormalities are already present at symptom onset, in a larger cohort and describe early signs in more detail.^14^ Part of the present cohort was previously published.^4^, ^6^, ^7^, ^14^, ^15^, ^16^, ^20^, ^25^, ^26^, ^27^, ^28^, ^29^, ^30^, ^31^, ^32^ Fumagalli et al. (2021) showed a natural history cohort of late‐infantile patients with low MLD MRI scores at baseline, substantiating our findings that MRIs of early‐onset MLD can be only mildly abnormal in early stages. In the Italian cohort, corpus callosum involvement was comparable to our results with splenial lesions in 18/21 (86%) or 6/8 (75%) when restricted to MRIs within 12 months after onset.^5^ Martin et al. (2012) described a symptomatic cohort of 10 late‐infantile and 3 early‐juvenile patients in whom corpus callosum involvement was present in 100% of the initial scans.^33^

There is a considerable risk of initial misdiagnosis in these symptomatic late‐infantile patients with normal or only mildly affected MRI scans. This is even more so when late‐infantile patients present with strabismus or severe sensorimotor polyneuropathy, sometimes manifesting after mild viral infections and temporarily improving with immunomodulatory therapy.^25^, ^34^, ^35^ Still, these initially mild MRIs may rapidly change into severely abnormal MRIs. The fast MRI deterioration corroborates earlier findings of Beerepoot et al. that those patients have high blood neurofilament light levels indicative for already present massive neuroaxonal damage.^29^

To better understand the absence of classic MRI abnormalities in this subgroup of symptomatic late‐infantile patients, we hypothesized that immature myelin may disguise clear abnormalities. We, therefore, analyzed disease duration and age at scan. We indeed found a significantly lower mean age at MRI, 1.9 versus 2.3, in symptomatic late‐infantile patients with MLD MRI scores 0–6 than in late‐infantile patients with MLD MRI scores >6 who were between 1.8 and 2.9 years old. This corroborates the previously reported breaking point of ~1.75 years of age to develop clear T2‐hyperintensities^16^ and our results confirm the hypothesis that T2‐hyperintensities typically emerge after a certain minimum age, including splenial involvement as typical early MRI sign for late‐infantile MLD, which also was only found in patients after that age. The possible influence of time after onset may, however, also play a role in this phenomenon. In our cohort, the patients with MLD MRI scores 0–6 had a significantly shorter time after onset, 0.4 versus 0.7 years, compared to the >6 group. It is therefore likely that the combination of younger age at scan and shorter time after onset explains the normal or only mildly affected scans in half of the symptomatic late‐infantile patients around the time of diagnosis.

Another possible explanation for the absence of clear MRI abnormalities in the symptomatic late‐infantile MLD group is that the main culprit for clinical symptoms at that stage is peripheral neuropathy. This was, however, not fully confirmed by our findings, as most of these patients did have clear evidence of CNS involvement as well. Therefore, the absence of clear MRI abnormalities cannot be explained by exclusive PNS involvement.

Among symptomatic patients, only late‐infantile and three early‐juvenile patients were found to have MLD MRI scores between 0–6. These three exceptional symptomatic early‐juvenile patients had a symptom onset between age 2.7–3.5 years, early for patients with early‐juvenile presentation, and two of them had a clear PNS dominant phenotype. The third patient of this subgroup presented with fine motor signs, but nerve conduction studies were not performed. These three early‐juvenile outliers might therefore be considered as borderline late‐infantile patients, because of their unusually early age at onset, PNS dominant phenotype, and mild MRI abnormalities.^25^

The variation in time before onset as well as the relatively low number of pre‐symptomatic patients did not allow us to make statements about typical characteristics of pre‐symptomatic MRIs. In spite of these limitations, the scans in pre‐symptomatic patients certainly confirmed that in early‐onset MLD the largest proportion of the MLD MRI score can be attributed to affected parieto‐occipital WM, whereas in late‐onset patients there is a frontal predominance.^14^, ^18^, ^33^, ^36^ This study has been unable to demonstrate that symptomatic adult patients have a predominantly affected frontal lobe, which can be explained by often late diagnosis and already extensive WM involvement, but also by the limitations of the MLD MRI scoring system.^18^ The items of the scoring system do not allow to, for example, rate local (frontal) atrophy, making the findings on regional distribution of abnormalities somewhat limited.

In the present study, we did not perform biochemical and genetic cross‐analysis with MRI findings. The interaction of arylsulfatase A activity or genotype^6^ with clinical presentation and thereby also MRI phenotype is complex and only partly understood.^37^ Cross‐center comparisons of arylsulfatase A enzyme activity and/or urinary sulfatides are currently not possible because of different methodologies and reference values.^4^ In addition, conventionally measured residual arylsulfatase A activity, using artificial substrates, only partially correlates with clinical phenotype.^4^, ^6^ Regarding *ARSA* variants, pathogenic variants resulting in completely inactive arylsulfatase A, so called null‐alleles, are associated with early‐onset MLD.^4^, ^37^ Detailed phenotypic predictions based on genotype can, however, not be made,^37^ and are part of other collaborative research projects.

In the future, other MRI modalities including diffusion‐weighted parameters, demyelination load, and MR‐spectroscopy may be of additional value in classifying nonspecific WM changes and establishing early diagnosis as they appear to be sensitive markers for damaged brain tissue.^15^, ^26^, ^33^ The use of gadolinium contrast may be helpful as well. Although demyelination in MLD is not accompanied by contrast enhancement on MRI, contrast enhancement of cranial nerves can be found in early stages of early‐onset MLD.^25^, ^35^, ^38^ Future prospective studies need to address the additional value of other MRI sequences in early diagnosis.

To conclude, in late‐infantile patients, clinical (CNS) signs may precede MRI abnormalities, whereas in juvenile and adult patients the MRI precedes clinical signs. Brain MRI in early‐onset MLD does not necessarily display evident WM abnormalities but resembles incomplete or delayed myelination. Even in early‐onset patients with developmental stagnation, CNS symptoms, or strabismus, the MRI may be (almost) normal. This implies that, as long as there is no newborn screening for MLD, laboratory confirmation should always be sought when MLD is suspected on clinical grounds, such as an early onset demyelinating polyneuropathy. Recognizing early MRI abnormalities and understanding their natural history is crucial in a field with ongoing clinical trials and emerging therapeutic options.

### Recommendations for clinicians

- Early‐onset MLD patients might have normal or non‐characteristic, mildly affected MRIs when already symptomatic.
- Elevated T2‐signal in the corpus callosum, centrum semiovale, or parieto‐occipital WM might be a first sign of MLD. In particular, in patients older than 2 years, this should be considered as abnormal and precedes symptom onset.
- CNS symptoms may precede MRI abnormalities in children with late‐infantile MLD. It is helpful to pay additional attention to the presence of CNS symptoms (signs of upper motor neuron involvement such as scissoring, Babinski signs, elevated muscle tone, and increased tendon reflexes) during physical examination. This will help to avoid misdiagnosis as an isolated peripheral demyelinating polyneuropathy.
- In adult MLD patients, dense T2‐hyperintensities and signs of atrophy are not necessarily accompanied by a tigroid pattern.

## Conflicts of Interest

BB is co‐founder, shareholder and CTO of AIRAmed GmbH (outside of current work). MSvdK is advisor and/or co‐investigator for trials in leukodystrophies (Ionis, Calico), without personal payment. She has a patent P112686CA00, therapeutic effects of Guanabenz treatment in vanishing white matter, assigned to the VU University Medical Center. NIW is advisor and/or co‐investigator for trials in Metachromatic Leukodystrophy and other leukodystrophies (Shire/Takeda, Orchard, Ionis, PassageBio, VigilNeuro, Sana Biotech), but receives no personal payment related to this role. SG received institutional research support from Shire/Takeda, outside of the submitted work. He is an advisor and co‐investigator for trials in Metachromatic Leukodystrophy (Shire/Takeda, Orchard, Bioclinica), but receives no personal payment related to this role.

Box 1

**Table:** 

Late‐infantile MLD (LI)	Manifesting symptoms <2.5 years of age
Early‐juvenile MLD (EJ)	Manifesting symptoms between 2.5–6 years of age
Late‐juvenile MLD (LJ)	Manifesting symptoms between 6–16 years of age
Adult MLD	Manifesting symptoms >16 years of age
Mild MRI	MLD MRI score ≤6
Moderate MRI	MLD MRI score ≥7 and ≤ 15
Severe MRI	MLD MRI score ≥ 16
Early MRI	MRI around the time of diagnosis, defined as <1.5 year after disease manifestation for late‐infantile, <2.5 years after disease manifestation for early‐juvenile and late‐juvenile, and <5.5 years after disease manifestation for adult patients.
Pre‐symptomatic (PS)	Having no symptoms at all, or an isolated peripheral polyneuropathy at ENG without clinical manifestation
Motor phenotype	Predominant motor symptoms (e.g. loss of motor skills, ataxia, spasticity, signs of peripheral polyneuropathy)
Cognitive phenotype	Predominant cognitive symptoms (e.g. learning difficulties, behavioral changes, psychiatric symptoms)
Mixed phenotype	Both motor and cognitive symptoms, no clear phenotype dominance
CNS dominant	Symptoms can predominantly be attributed to CNS involvement (e.g. spasticity, hyperreflexia, scissoring, Babinski sign, cognitive decline)
PNS dominant	Symptoms can predominantly be attributed to PNS involvement (e.g. muscle weakness, hyporeflexia, hypoesthesia)
GMFC‐MLD	Gross Motor Function Classification for MLD

## Supporting information


**Table S1.** Case summaries.
