# Supplementary material for: Recognizing early MRI signs (or their absence) is crucial in diagnosing metachromatic leukodystrophy
Source: Ann Clin Transl Neurol. 2022 Nov 5;9(12):1999–2009. doi: 10.1002/acn3.51692 (PMC9735365; doi:10.1002/acn3.51692)
Supplement: Supplementary file 1 — Table S1. Case summaries. [file ACN3-9-1999-s001.docx]

### Supplementary material

**Supplementary table 1** - Case summaries

*Pre-symptomatic cases were usually identified through family screening after an affected sibling was diagnosed.

|  | | | Sex | | | Age at onset | Age at scan | MLD MRI score | GMFC-MLD score at the time of MRI | Motor. cognitive or mixed phenotype at onset | PNS or CNS dominant phenotype at onset | Presence of CNS symptoms |
| --- | --- | --- | --- | --- | --- | --- | --- | --- | --- | --- | --- | --- |
| Late-infantile | Symptomatic | 1 | male | | | 1..5 | 2.3 | 3 | 6 | Motor phenotype | PNS | yes |
|  |  | 2 | female | | | 1..3 | 2..3 | 13 |  | Motor phenotype | CNS | yes |
|  |  | 3 | male | | | 1..0 | 2..2 | 5 | 2 | Motor phenotype | PNS | yes |
|  |  | 4 | female | | | 1.8 | 2.0 | 13 | 6 | Motor phenotype | CNS | yes |
|  |  | 5 | female | | | 1.0 | 2.2 | 11 | 6 | Motor phenotype | PNS | yes |
|  |  | 6 | female | | | 1.6 | 1.9 | 3 | 1 | Motor phenotype | PNS | yes |
|  |  | 7 | male | | | 1.0 | 2.1 | 2 | 2 | Motor phenotype | PNS | no |
|  |  | 8 | female | | | 1.3 | 2.3 | 13 | 3 | Motor phenotype | PNS | yes |
|  |  | 9 | male | | | 1.9 | 2.4 | 13 | 4 | Motor phenotype | CNS | yes |
|  |  | 10 | male | | | 1.8 | 2.4 | 8 | 5 | Motor phenotype | PNS | yes |
|  |  | 11 | female | | | 1.0 | 2.3 | 10 | 3 | Motor phenotype | PNS | yes |
|  |  | 12 | male | | | 1.3 | 1.4 | 3 | 2 | Motor phenotype | CNS | yes |
|  |  | 13 | female | | | 2.0 | 2.9 | 8 | 2 | Motor phenotype | PNS | yes |
|  |  | 14 | female | | | 1.8 | 2.3 | 10 | 2 | Mixed phenotype | PNS | yes |
|  |  | 15 | male | | | 1.5 | 2.1 | 12 | 2 | Motor phenotype | PNS | yes |
|  |  | 16 | male | | | 1.4 | 1.8 | 5 | 3 | Motor phenotype | CNS | yes |
|  |  | 17 | female | | | 2.4 | 2.4 | 3 | 2 | Motor phenotype | CNS | yes |
|  |  | 18 | male | | | 2.0 | 2.0 | 3 | 1 | Motor phenotype |  | yes |
|  |  | 19 | male | | | 1.4 | 1.7 | 3 | 2 | Motor phenotype | CNS | yes |
|  |  | 20 | male | | | 2.5 | 2.2 | 3 | 2 | Motor phenotype | CNS | yes |
|  |  | 21 | male | | | 1.4 | 2.3 | 14 | 6 | Motor phenotype | CNS | yes |
|  |  | 22 | male | | | 2.0 | 2.8 | 10 | 5 |  |  |  |
|  |  | 23 | female | | | 1.7 | 1.5 | 0 | 0 | Motor phenotype |  | yes |
|  |  | 24 | female | | | 2.3 | 2.3 | 12 | 3 | Motor phenotype | CNS | yes |
|  |  | 25 | male | | | 1.4 | 1.9 | 3 | 2 | Motor phenotype | PNS | no |
|  |  | 26 | male | | | 1.0 | 1.5 | 1 | 2 | Motor phenotype |  |  |
|  |  | 27 | female | | | 1.8 | 2.1 | 10 | 1 | Motor phenotype | PNS | yes |
|  |  | 28 | male | | | 1.4 | 2.3 | 11 | 2 | Motor phenotype | CNS | yes |
|  |  | 29 | female | | | 2.0 | 2.3 | 16 | 2 |  |  |  |
|  |  | 30 | male | | | 1.0 | 1.8 | 2 | 3 |  |  |  |
|  |  | 31 | male | | | 1.0 | 1.8 | 10 | 2 | Motor phenotype | PNS | yes |
|  |  | 32 | male | | | 1.3 | 1.2 | 2 | 3 | Motor phenotype |  |  |
|  |  | 33 | male | | | 1.3 | 1.7 | 0 | 1 | Motor phenotype | PNS | no |
|  |  | 34 | female | | | 1.5 | 1.9 | 8 | 6 | Motor phenotype | CNS | yes |
|  |  | Total | | N | 34 | 34 | 34 | 34 | 33 | 31 | 27 | 29 |
|  | Pre-symptomatic | 1 | male | | |  | 0.4 | 0 | 0 |  |  |  |
|  |  | 2 | female | | | 2.3 | 1.8 | 0 | 0 |  |  |  |
|  |  | 3 | female | | |  | 0.5 | 0 | 0 |  |  |  |
|  |  | 4 | female | | |  | 0.5 | 0 | 0 |  |  |  |
|  |  | 5 | female | | |  | 1.8 | 4 | 0 |  |  |  |
|  |  | 6 | female | | | 1.5 | 0.8 | 2 | 0 |  |  |  |
|  |  | 7 | female | | |  | 1.6 | 1 | 0 |  |  |  |
|  |  | 8 | male | | |  | 2.1 | 3 | 0 |  |  |  |
|  |  | 9 | female | | |  | 1.7 | 0 | 0 |  |  |  |
|  |  | Total | | N | 9 | 2 | 9.0 | 9 | 9 |  |  |  |
|  | Total | N | 43 | | | 36 | 43 | 43 | 42 | 31 | 27 | 29 |
| Early-juvenile | Symptomatic | 1 | male | | | 3.0 | 4.7 | 4 | 1 | Motor phenotype | PNS | no |
|  |  | 2 | male | | | 5.0 | 7.2 | 19 | 1 | Mixed phenotype | CNS | yes |
|  |  | 3 | male | | | 6.0 | 6.6 | 14 | 1 | Mixed phenotype | PNS | yes |
|  |  | 4 | male | | | 6.0 | 6.6 | 14 | 1 | Mixed phenotype | PNS | yes |
|  |  | 5 | male | | | 5.0 | 6.8 | 14 | 1 | Mixed phenotype | CNS | yes |
|  |  | 6 | female | | | 4.0 | 5.8 | 19 | 1 | Motor phenotype | PNS |  |
|  |  | 7 | female | | | 4.0 | 6.1 | 14 | 1 | Motor phenotype | CNS | yes |
|  |  | 8 | male | | | 5.0 | 6.5 | 15 | 1 | Mixed phenotype | CNS | yes |
|  |  | 9 | male | | | 4.5 | 5.4 | 14 | 1 | Mixed phenotype | CNS | yes |
|  |  | 10 | female | | | 5.0 | 7.1 | 13 | 1 | Motor phenotype | CNS | yes |
|  |  | 11 | male | | | 4.0 | 5.6 | 18 | 1 | Motor phenotype | CNS | yes |
|  |  | 12 | male | | | 4.0 | 6.3 | 18 | 1 | Motor phenotype | CNS | yes |
|  |  | 13 | male | | | 3.5 | 4.2 | 4 | 0 |  |  |  |
|  |  | 14 | male | | | 3.6 | 3.7 | 19 | 1 |  |  |  |
|  |  | 15 | male | | | 2.7 | 2.9 | 1 | 1 | Motor phenotype | PNS | yes |
|  |  | 16 | female | | | 5.3 | 5.7 | 20 | 1 | Mixed phenotype | CNS | yes |
|  |  | Total | | N | 16 | 16 | 16 | 16 | 16 | 14 | 14 | 13 |
|  | Pre-symptomatic | 1 | female | | |  | 4.6 | 8 | 0 |  |  |  |
|  |  | 2 | female | | |  | 2.2 | 2 | 0 |  |  |  |
|  |  | 3 | male | | |  | 4.3 | 6 | 0 |  |  |  |
|  |  | 4 | female | | |  | 7.0 | 0 | 0 |  |  |  |
|  |  | 5 | male | | |  | 1.4 | 0 | 0 |  |  |  |
|  |  | 6 | male | | | 5.3 | 4.2 | 12 | 0 |  |  |  |
|  |  | 7 | male | | | 5.3 | 4.2 | 13 | 0 |  |  |  |
|  |  | 8 | female | | |  | 0.9 | 0 | 0 |  |  |  |
|  |  | Total | | N | 8 | 2 | 8.0 | 8 | 8 |  |  |  |
|  | Total | N | 24 | | | 18 | 24 | 24 | 24 | 14 | 14 | 13 |
| Late-juvenile | Symptomatic | 1 | female | | | 7.0 | 8.5 | 20 | 1 | Mixed phenotype | CNS | yes |
|  |  | 2 | female | | | 14.0 | 15.8 | 20 | 1 | Mixed phenotype | CNS | yes |
|  |  | 3 | female | | | 6.0 | 7.0 | 18 | 1 | Motor phenotype | CNS | yes |
|  |  | 4 | male | | | 6.0 | 7.0 | 14 | 1 | Motor phenotype | CNS | yes |
|  |  | 5 | female | | | 13.0 | 15.1 | 21 | 1 | Cognitive phenotype | CNS | yes |
|  |  | 6 | female | | | 7.0 | 7.1 | 17 | 1 | Motor phenotype | PNS |  |
|  |  | 7 | female | | | 10.5 | 12.4 | 17 | 1 | Mixed phenotype | CNS | yes |
|  |  | 8 | male | | | 12.0 | 13.8 | 12 | 0 | Mixed phenotype | CNS | yes |
|  |  | 9 | male | | | 9.0 | 9.3 | 18 | 2 | Motor phenotype | CNS | yes |
|  |  | 10 | male | | | 7.0 | 8.5 | 18 | 1 | Mixed phenotype | CNS | yes |
|  |  | 11 | female | | | 6.0 | 6.8 | 19 | 1 | Motor phenotype | CNS | yes |
|  |  | 12 | female | | | 12.0 | 14.4 | 11 | 1 | Mixed phenotype | PNS |  |
|  |  | Total | | N | 12 | 12 | 12 | 12 | 12 | 12 | 12 | 10 |
|  | Pre-symptomatic | 1 | female | | | 9.3 | 8.8 | 16 | 0 |  |  |  |
|  |  | 2 | female | | | 12.4 | 9.4 | 3 | 0 |  |  |  |
|  |  | 3 | male | | |  | 13.9 | 7 | 0 |  |  |  |
|  |  | 4 | female | | |  | 14.5 | 11 | 0 |  |  |  |
|  |  | 5 | male | | | 12.0 | 9.3 | 0 | 1 |  |  |  |
|  |  | 6 | female | | |  | 6.4 | 0 | 0 |  |  |  |
|  |  | 7 | male | | |  | 17.8 | 10 | 0 |  |  |  |
|  |  | 8 | female | | |  | 7.4 | 4 | 0 |  |  |  |
|  |  | Total | | N | 8 | 3 | 8.0 | 8 | 8 |  |  |  |
|  | Total | N | 20 | | | 15 | 20 | 20 | 20 | 12 | 12 | 10 |
| Adult | Symptomatic | 1 | male | | | 25.0 | 28.3 | 12 | 0 | Cognitive phenotype | CNS | yes |
|  |  | 2 | female | | | 25.0 | 29.1 | 24 |  | Cognitive phenotype | CNS | yes |
|  |  | 3 | male | | | 18.0 | 19.3 | 22 | 0 | Cognitive phenotype | CNS | yes |
|  |  | 4 | male | | | 25.0 | 30.3 | 23 |  | Cognitive phenotype | CNS | yes |
|  |  | 5 | female | | | 20.0 | 22.2 | 19 | 1 | Cognitive phenotype | CNS | yes |
|  |  | 6 | female | | | 18.0 | 19.2 | 26 | 1 | Mixed phenotype | CNS | yes |
|  |  | 7 | male | | | 34.0 | 36.3 | 21 | 1 | Mixed phenotype | CNS | yes |
|  |  | 8 | female | | | 28.0 | 33.0 | 17 | 1 | Mixed phenotype | CNS | yes |
|  |  | 9 | male | | | 21.0 | 22.0 | 21 | 0 | Cognitive phenotype | CNS | yes |
|  |  | 10 | female | | | 19.0 | 23.0 | 26 | 0 | Cognitive phenotype | CNS | yes |
|  |  | 11 | male | | | 17.0 | 17.0 | 20 | 2 | Mixed phenotype | CNS | yes |
|  |  | 12 | female | | | 33.0 | 35.0 | 20 | 0 | Cognitive phenotype | CNS | yes |
|  |  | Total | | N | 12 | 12 | 12 | 12 | 10 | 12 | 12 | 12 |
|  | Pre-symptomatic | 1 | female | | |  | 35.3 | 10 | 0 |  |  |  |
|  |  | 2 | female | | |  | 19.2 | 7 | 0 |  |  |  |
|  |  | 3 | female | | |  | 26.6 | 19 | 0 |  |  |  |
|  |  | 4 | female | | |  | 25.3 | 12 | 0 |  |  |  |
|  |  | 5 | male | | |  | 23.0 | 12 | 1 |  |  |  |
|  |  | Total | | N | 5 |  | 5 | 5 | 5 |  |  |  |
|  | Total | N | 17 | | | 12 | 17 | 17 | 15 | 12 | 12 | 12 |
| Total |  | | 104 | | | 81 | 104 | 104 | 101 | 69 | 65 | 64 |
